# Kawasaki Disease and the Use of the Rotavirus Vaccine in Children: A Systematic Review and Meta-Analysis

**DOI:** 10.3389/fphar.2019.01075

**Published:** 2019-09-24

**Authors:** Natália Gibim Mellone, Marcus Tolentino Silva, Mariana Del Grossi Paglia, Luciane Cruz Lopes, Sílvio Barberato-Filho, Fernando de Sá Del Fiol, Cristiane de Cássia Bergamaschi

**Affiliations:** ^1^Pharmaceutical Science Graduate Course, University of Sorocaba, Sorocaba, Brazil; ^2^Federal University of Amazonas, Faculty of Medicine, Manaus, Brazil

**Keywords:** Kawasaki disease, rotavirus vaccine, safety, systematic review, adverse effect

## Abstract

**Background:** The vaccine against the rotavirus is an effective measure in reducing hospitalizations and mortality caused by the virus. However, its use can result in serious adverse effects. The available evidence on Kawasaki disease has not yet been reported in the literature. This study investigated the risk of developing Kawasaki disease with the use of rotavirus vaccines in children.

**Methods:** This is a systematic review of data collected from studies retrieved on the following databases: Cochrane, MEDLINE, Embase, CINAHL, Scopus, Web of Science, HealthSTAR, Lilacs, Clinical trial.gov, and International Clinical Trials Registry Platform, up to the 15^th^ of August 2018, with no restrictions on language or date of publication. The outcomes measured were incidence of Kawasaki disease, risk of developing the disease, and rate of discontinuation of the vaccination schedule. Four reviewers independently selected the studies, performed data extraction, and assessed the quality of evidence. A meta-analysis of random effects was performed.

**Results:** A total of 13 publications were included, with a population of 164,434 children included in the meta-analysis. The incidence of Kawasaki disease (24 cases per 100,000, 95% CI = 11.98–48.26) in the vaccinated children was low. No difference between the vaccines was found in the prevalence rate of adverse effects (RR = 1.55, 95% CI = 0.41–5.93). Use of the vaccines was not associated with risk of developing Kawasaki disease (low-quality evidence). None of the studies reported the rate of discontinuation of the vaccination schedule.

**Conclusions:** The vaccines were associated with a low incidence of developing Kawasaki disease, showing no association with this serious adverse effect.

## Introduction

The rotavirus is the leading cause of severe diarrhea in infants and children worldwide, particularly in developing countries representing one of the main causes of morbidity in young children globally (Uhlig et al., 2014; Yin et al., 2015). The deaths caused by gastroenteritis due to infection by the rotavirus have been high in low-to-medium income Latin American countries (Bryce et al., 2005) and the Caribbean (Linhares et al., 2011).

Two vaccines are recommended by the World Health Organization (WHO) for use against the rotavirus, referred to as the pentavalent vaccine and the monovalent vaccine, introduced into immunization programs of some countries from 2006 onwards (Uhlig et al., 2014; World Health Organization, 2014). Other vaccines are commercially available, such as the oral monovalent Lanzhou lamb rotavirus in China (Yin et al., 2015), the monovalent Rotavin-M vaccine in Vietnam, and the monovalent Rotavac vaccine in India (Kollaritsch et al., 2015).

In 2009, and again in 2013, the WHO recommended the introduction of one of these vaccines into all national immunization programs (Soares-Weiser et al., 2012). The vaccines are administered *via* the oral route in babies, in two doses for the monovalent vaccine and three for the pentavalent vaccine. The monovalent vaccine is administered at between the 6th and 15th week of life while the pentavalent vaccine is given as a three-dose series between the 6th and 32nd week (Shatsky and Vaccine, 2006).

The most common adverse reactions associated with the use of the vaccine are cough, nasal discharge, diarrhea, irritability, loss of appetite, fever, and vomiting (Bravo et al., 2014). However, its use can also cause serious adverse effects, such as intussusception (Maglione et al., 2014) and Kawasaki disease (Soares-Weiser et al., 2012).

Kawasaki disease was included as a serious adverse effect in the package insert of the pentavalent vaccine after being reviewed by the manufacturer and approved by the Food and Drug Administration (FDA) in 2007, when a pre-licensure clinical trial revealed the presence of this effect in children after use of the vaccine (Hua et al., 2009).

Kawasaki disease has features compatible with common viral infections and mainly affects children, where almost 100% of cases occur in children younger than 5 years and genetically predisposed individuals (Makino et al., 2018). Children of Japanese and Asian-Pacific Island descent have the highest rates, and males have higher rates than females (Fuller, 2019).

In Asian countries, the incidence rates of Kawasaki disease are high (Singh et al., 2015). Recent Japanese data revealed the highest global annual incidence of disease in children under 5 years of age (Makino et al., 2018), with second and third highest rates reported for South Korea (Kim et al., 2014) and Taiwan (Lin et al., 2015), respectively. However, other Asian countries have a steadily increasing incidence of this disease (Saundankar et al., 2014; Wu et al., 2017). In Canada (Lin et al., 2010), the United States (Uehara and Belay, 2012), and Europe (Salo et al., 2012; Jakob et al., 2016), Kawasaki disease rates are significantly lower. The marked differences in incidence rates among different ethnicities strongly support the idea of a strong genetic basis of susceptibility (Holman et al., 2010).

The disease presents a variety of signs and symptoms, such as persistent fever for longer than 5 days, non-exudative bilateral conjunctivitis, erythema of the lips and oral mucosa, swelling of the extremities, cutaneous eruption, gastrointestinal symptoms, and lymphadenopathy. Aneurysm of the coronary artery or ectasia can develop in 20–25% of cases, along with other complications in untreated children, where the condition may evolve to myocardial infarction, ischemic heart disease, and death (Abrams et al., 2015).

Although most epidemiologic and immunologic evidence suggests that an infectious agent causes Kawasaki disease (Geier et al., 2008), this is not conclusive. Besides, the infectious agent and the genetic characteristics of susceptible children have yet to be elucidated (Principi et al., 2013); it is possible that vaccination play some role in the pathogenesis of Kawasaki disease (Burgner and Harnden, 2005; Rowley and Shulman, 2007). The distinctive immune system characteristics of children with Kawasaki disease could suggest that they respond to all antigenic stimulations, including those due to vaccines (Esposito et al., 2016) in a way that differs from that observed in healthy children. However, the motive, based on biochemical and immunological mechanisms, by which the rotavirus vaccines leads to Kawasaki disease, were not found in the literature.

The available evidence on risk of Kawasaki disease with the use of rotavirus vaccines has not yet been reported in the literature. This knowledge can help guide health professionals in clinical decision-making. This systematic review sought to answer the following PICO question: “what is the risk of Kawasaki disease in children who made use of rotavirus vaccines compared to those who did not?” Therefore, the objective of this study was to investigate the risk of developing Kawasaki disease with the use of rotavirus vaccines in children.

## Methods

### Protocol and Registration

The systematic review was performed according to the recommendations specified in the Cochrane Manual of Interventionist Reviews and reported according to the Preferred Reporting Items for Systematic Reviews and Meta-Analyses (PRISMA) checklist (Liberati et al., 2009; Moher et al., 2010; Higgins JPT, 2011)

This protocol was registered on the International Prospective Register of Systematic Reviews (PROSPERO: CRD4201604633, https://www.crd.york.ac.uk/prospero/display_record.php?RecordID=46334).

### Eligibility Criteria

#### Inclusion Criteria

This study included randomized clinical trials (RCT) and quasi-randomized and observational studies (case report, ecological, case series, adverse event report, cross-sectional, case-control, and cohort studies) involving children up to 32 weeks of age in use of vaccines (monovalent or pentavalent) against rotavirus.

#### Exclusion Criteria

Abstracts published in congresses not providing data on the incidence of the adverse effect were excluded.

### Search for Primary Studies

#### Electronic Searches

The following electronic databases were searched: Cochrane Central Register of Controlled Trials (CENTRAL), MEDLINE (*via* Ovid), Embase, Cumulative Index to Nursing and Allied Health Literature (CINAHL), Web of Science, HealthSTAR (*via* Ovid), Scopus, LILACS, Clinical trial.gov, and International Clinical Trials Registry Platform, up to the 15^th^ of August 2018, with no restrictions on language or date of publication.

#### Other Search Resources

The references from the eligible studies, the systematic reviews on the rotavirus vaccine, and FDA data were reviewed to identify other eligible studies. The National Health Surveillance Agency in Brazil and the National Brazilian Immunization Program were contacted by email *via* the individuals in charge to check for the existence of reports of Kawasaki disease associated with the vaccines. Information was requested for notifications of the disease as well as related signs and symptoms. Google Scholar (to find unindexed journals), ProQuest Dissertation and Theses Database, the Brazilian Digital Library of Thesis and Dissertations, and the Thesis and Dissertation Catalog of Coordenação de Aperfeiçoamento de Pessoal de Nível Superior (CAPES) were also consulted.

#### Search Strategies

The following Mesh descriptors and their combinations (entry terms) were used: rotavirus vaccines (or vaccines, rotavirus) and mucocutaneous lymph node syndrome (or Kawasaki syndrome or lymph node syndrome or mucocutaneous, or Kawasaki disease) for the article search. Search strategies for each database used are available in **Supplementary Data Sheet 1**.

#### Outcomes Assessed

The primary outcome was the incidence of Kawasaki disease associated with the vaccines (number of cases of the disease/total number of children vaccinated for rotavirus). The secondary outcomes were risk of Kawasaki disease comparing each rotavirus vaccine with a control group, and the rate of discontinuation of the vaccination schedule. Given the lack of a standard case definition for the disease, the diagnostic criteria adopted by the studies was used.

The rate of adverse effects was expressed according to the following categories: very common ≥1/10 (≥10%), common ≥1/100 and <1/10 (≥1% and <10%), uncommon ≥1/1,000 and <1/100 (≥0.1% and <1%), rare ≥1/10,000 and <1/1,000 (≥0.01% and <0.1%), and very rare <1/10,000 (<0.01%) (Meyboom and Egberts, 1999).

#### Study Selection

The reviewers (NM and CB), working independently, selected the potentially relevant studies and applied the eligibility criteria. The full texts of all the potentially eligible articles were obtained and then the reviewers (NGB and MP, FSD-F, and SB-F) assessed the eligibility of each full article. Disagreements were resolved by consensus and, when necessary, submitted to a third reviewer (LL). The Endnote X7 software package was employed for study selection.

#### Data Extraction

The data were extracted by all reviewers, working independently, using a data extraction form. In the case of articles published only as abstracts or those with key information missing, their respective authors were contacted to obtain the necessary information. Disagreements were again resolved by consensus and, when necessary, submitted to a third reviewer.

Information was collected on: i) the characteristics of the studies (objectives, study design, country where the study was conducted, type of vaccine, data collection period, and conclusions) and ii) the study population (age, gender, race, sample size [number of children vaccinated]), vaccination status (doses of rotavirus vaccine given to child prior to onset of Kawasaki disease), diagnostic method for Kawasaki’s disease, time to disease onset after vaccination (days of rotavirus vaccination until the cases developed the disease), and other concomitant vaccinations, when available.

Subgroup analyses were proposed for age, gender, race, and country, where applicable. The heterogeneity of the studies was determined using the χ^2^ test and I^2^ statistic. The following heterogeneity was considered: 0–25% (low heterogeneity), 50% (moderate heterogeneity), and 75% (high heterogeneity) (Higgins et al., 2003).

#### Assessment of Risk of Bias

The quality of the observational studies was determined using the tool described by Munn et al. (2014). This step was performed by all reviewers, working in pairs and independently. This tool includes 10 items for critical assessment of the methodological quality of prevalence studies. For each criterion, the study was attributed “yes” or “no” or “not applicable.” The total number of “yes” answers per study was tallied. A higher number of “yes” answers indicates a lower risk of bias of the study. The risk of bias of clinical trials was that reported by the systematic review (Soares-Weiser et al., 2012), which used the Cochrane risk of bias tool to assess the following criteria: sequence; allocation concealment; blinding of the patient, healthcare professionals, outcome assessors, data collectors, and data analysts; incomplete outcome data; selective outcome reporting; and major baseline imbalance.

#### Data Synthesis

The random-effect meta-analysis was performed using the STATA software package (version 14.2) (Montori et al., 2008). Given that this was a systematic review of adverse effect, RCTs and cohort studies were included in the meta-analysis, where both study designs allow information on adverse effects to be collected. Data were summarized according to incidence of the disease per 100,000 vaccinated children and relative risk (RR) with a 95% confidence interval (95% CI). When the meta-analysis was not suitable, a narrative summary of the studies was provided.

#### Quality of Evidence

The quality of evidence of the studies was assessed using the Grading of Recommendations Assessment, Development, and Evaluation (GRADE) approach (Guyatt et al., 2011). In this approach, RCTs start with high-quality evidence but can be assessed by one or more of the five categories of limitation of the studies: risk of bias, inconsistency, indirect measurement, imprecision, and publication bias. Observational studies start with low-quality evidence, which can increase according to the assessment of the categories.

## Results

### Literature Search Results

**Figure 1:**
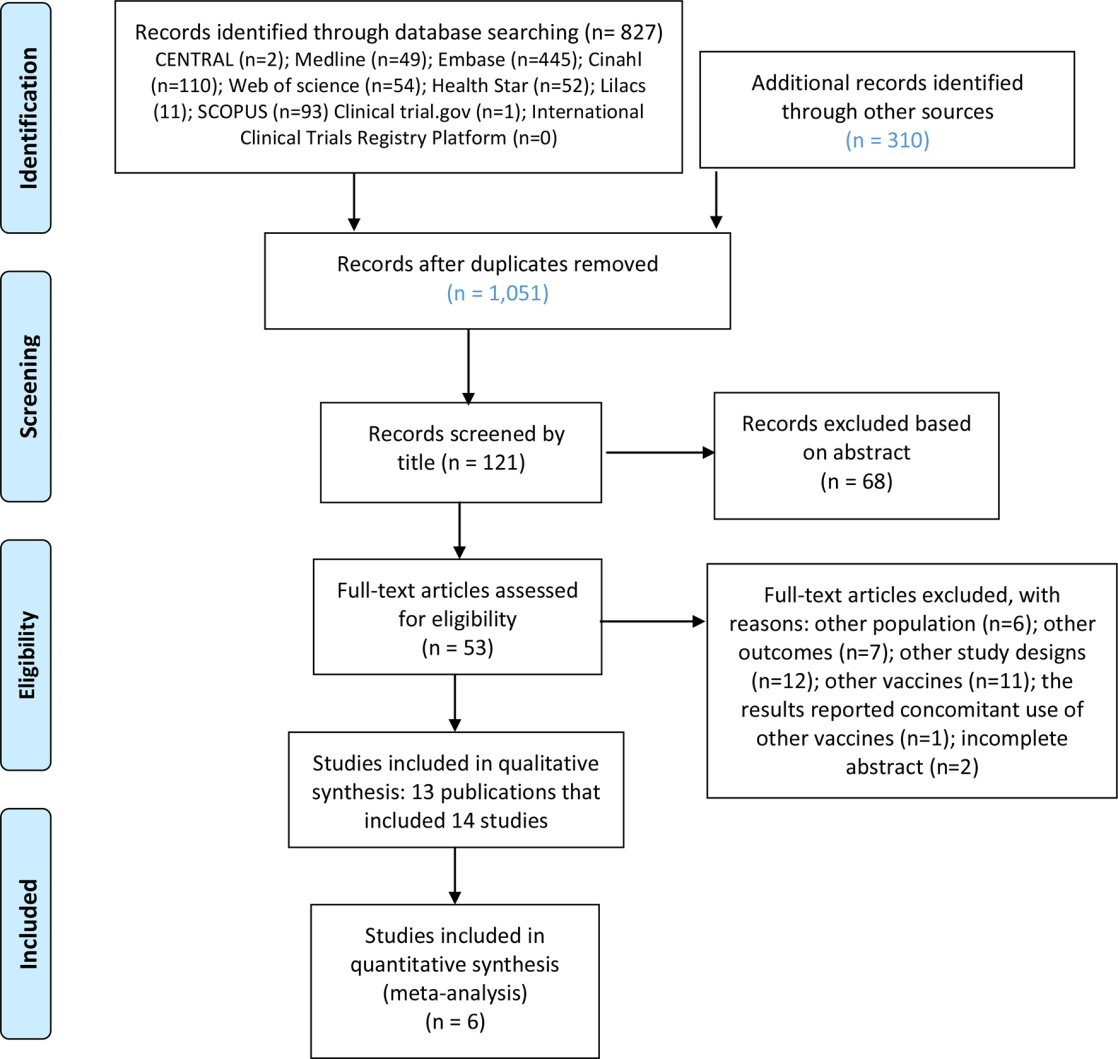
Study selection process.

A total of 1,051 publications were revised (after duplicates removed) and 52 potential eligible publications selected. Of these articles, 13 publications (total 14 studies) were included. One of the publications, a package inserts by the FDA, contained information on two studies: a phase III trial and a phase IV study (**Figure 1**). There were no Brazilian studies or notifications reported in Brazil on Kawasaki disease due to use of the rotavirus vaccines. A search for articles in the references of the systematic review (Soares-Weiser et al., 2012) led to the identification of a further three clinical trials (Phua et al., 2005; Phua et al., 2009; Salinas et al., 2005), which were subsequently included in the study.

### Description of Studies Included in Narrative Syntheses

The characteristics and outcomes of the studies included are given in **Table 1** (descending order of year of publication). This review found information in: two case reports (Uhlig et al., 2014; Chang and Islam, 2018), four cohort studies (Belongia et al., 2010; Loughlin et al., 2012; Layton et al., 2018; RotaTeq, 2017), one cross-sectional study (Oberle et al., 2010), three adverse event reports (Geier et al., 2008; Hua et al., 2009; Paulke-Korinek et al., 2013), and four RCTs (Phua et al., 2005; Salinas et al., 2005; Phua et al., 2009; RotaTeq, 2017).

**Table 1:** Characteristics of the studies and outcomes found in children vaccinated against the rotavirus.

Author (year)	Study design	Data collection period	Country	Vaccines	Diagnostic methods of KD	N of KD cases	Total (N of children vaccinated)	Total of children with AE	OR or RR (95% CI)
Chang and Islam (2018)	Case report	NR	USA	RV1	Standard (AHA)	1	1	1	Not applicable
Layton et al. (2018)	Cohort	2006–2014	USA	RV1 and RV5	ICD	23	2,468,002	NR	RR = 0.54 (0.20–1.48)
Yin et al. (2015) (Park et al., 2011)	Case report	2015	China	LLR	NR	1	1	1	Not applicable
Paulke-Korinek et al. (2013)	Adverse event reports	2010–2011	Austria	RV1 and RV5	NR	0	NR	823	Not applicable
Loughlin et al. (2012)	Cohort	2006–2007	USA	RV5	ICD	3	85,150	NR	RR = 0.4 (0.01–8.47)
Belongia et al. (2010)	Cohort	2006–2008	USA	RV5	Medical record	16	207,621	NR	OR = 0.28 (0.07–1.09)
Oberle et al. (2010)	Cross-sectional	2001–2009	Germany	RV5	Medical record	4	NR	1.088	OR = 3.1 (1.1–9.1)
Information from package insert (phase III trial) (M. U.S. Food and Drug Administration)	RCT	NR	NR	RV5	NR	5	36,150	NR	RR = 4.9 (0.6–239.1)
Information from package insert (phase IV study) (M. U.S. Food and Drug Administration)	Cohort	NR	NR	RV5	NR	1	17,433	NR	RR = 0.7 (0.01–55.56)
Hua et al. (2009)	Adverse event reports	1990–2007	USA	RV5	Medical records	16	NR	239,535	NR
Phua et al. (2009)	RCT	2003–2005	Hong Kong, Singapore, and Taiwan	RV1	NR	1	4,272	NR	RR = 2.9 (0.12–72.83)
Geier et al. (2008)	Adverse event reports	2006–2007	USA	RV5	NR	16	NR	1,526	NR
Phua et al. (2005)	RCT	2001–2003	Singapore	RV1	NR	2	1,811	NR	RR = 1.8 (0.09–37.53)
Salinas et al. (2005)	RCT	2001–2003	Brazil, Mexico, and Venezuela	RV1	NR	1	1,618	NR	RR = 1.0 (0.04–24.44)

USA, United States of America; AHA, American Heart Association; KD, Kawasaki disease; AE, adverse effect; OR, odds ratio; RR, relative risk; 95% CI, 95% confidence interval; NR, not reported; RV1, monovalent vaccine; RV5, pentavalent vaccine; ICD, International Classification of Disease; LLR, Lanzhou lamb rotavirus; RCT, randomized clinical trial.

One case report described a 4-month-old Caucasian child, presenting classic Kawasaki disease shortly after receiving vaccines (pneumococcal 13, diphtheria–tetanus–pertussis, polio, Hepatitis B, and rotavirus vaccine). The case supports that vaccines may be associated with vasculitis and Kawasaki disease and that ongoing, systematic surveillance of such events is warranted (Chang and Islam, 2018).

Only one study reported the use of the Lanzhou lamb rotavirus vaccine. It consisted of a clinical case report of a 20-month-old Chinese child with Kawasaki disease after use of the rotavirus vaccine concomitantly with the hepatitis A vaccine. According to the authors, the rotavirus vaccine may have played a key role in the development of the disease, but no causal relationship between the effect and the vaccine could be established based on a single case (Yin et al., 2015).

A cohort study of commercial insurance data investigated the risk of adverse events associated with rotavirus vaccines. Most children received a concomitant diphtheria–tetanus–pertussis vaccine. Of a total of 2,468,002 vaccines, 23 cases of Kawasaki disease were found (Layton et al., 2018).

An adverse event report study determined the prevalence of adverse events associated with the monovalent and pentavalent vaccines using the sentinel surveillance system. A total of 833 adverse events were associated with the vaccines, but no cases of Kawasaki disease were reported (Paulke-Korinek et al., 2013).

The cohort study identified post-marketing adverse effects after routine use of the pentavalent vaccine from records held on an electronic database. Of the 85,150 children that received the rotavirus vaccine, there were 3 cases of Kawasaki disease in the intervention group (only 1 case was confirmed to have occurred within 30 days of vaccination) and 1 in the control group (recipients of diphtheria–tetanus–acellular pertussis vaccine). No increased risk of developing the disease due to the vaccine was observed (Loughlin et al., 2012).

Another cohort study assessed the risk of intussusception and other adverse events using the Vaccine Adverse Event Reporting System (VAERS) database among children aged 4–48 weeks who received the pentavalent vaccine (intervention group) compared to a non-exposed group. Similarly, no association of developing Kawasaki disease with use of the pentavalent vaccine was found (Geier et al., 2008).

A cross-sectional study used the database of the Paul Ehrlich Institute to assess the cases of Kawasaki disease associated with the use of monovalent and pentavalent vaccines (between 2001 and 2010). Four cases of the disease were reported from a total of 1,088 adverse events associated with the use of the pentavalent vaccine in children aged 2–5 months. The study reported the day on which the effect occurred (average of 6.3 days after vaccination). Three children were in use of another vaccine concomitantly (Oberle et al., 2010).

An adverse event report study assessed the data on adverse events associated with the pentavalent vaccine using the VAERS database. A total of 1,526 adverse events were associated with the pentavalent vaccine. Of 16 children found to have Kawasaki disease, 5 used this vaccine (Belongia et al., 2010).

Another study also searched the VAERS information to identify Kawasaki disease in children following use of vaccines with licensure in the United States. The authors identified a total of 239,535 events, including 107 cases of Kawasaki disease, 16 of which were following the use of the pentavalent vaccine. The study did not specify the number of pentavalent vaccines administered (Hua et al., 2009).

The package inserts by the FDA for the pentavalent vaccine described the results of two studies (a post-marketing study—phase IV and phase III clinical trials—the Rotavirus Efficacy Safety Trial/REST). The cohort study compared the data on the disease in 17,433 children who received the pentavalent vaccine *versus* a control group of 12,339 that received a diphtheria, tetanus, and pertussis vaccine, revealing only one case of Kawasaki disease within 30 days of vaccination (RotaTeq, 2017).

The clinical trial found five cases of Kawasaki disease in 36,160 vaccinated children and one case in 35,536 children who received a placebo (42 days after the use of the pentavalent vaccine). The other three clinical trials also found no association of the adverse effect with the use of the monovalent vaccine (Phua et al., 2005; Phua et al., 2009; Salinas et al., 2005).

### Risk of Bias of the Studies

The cohort studies had risk of bias, having failed to account for possible confounding factors and/or to perform subgroup analyses. The cross-sectional and adverse event report studies had shortcomings for a larger number of assessed criteria that included the problems observed in the cohort studies plus those of data analysis with problems of underestimation of prevalence data for Kawasaki disease (**Table 2**).

**Table 2:** Risk of bias of observational studies according to criteria adopted by Munn et al. (2014).

Study author and year	Was the sample representative of the target population?	Were study participants recruited in an appropriate way?	Was the sample size adequate?	Were the study subjects and setting described in detail?	Was the data analysis conducted with sufficient coverage of the identified sample?	Were objective, standard criteria used for measurement of the condition?	Was the condition measured reliably?	Was there appropriate statistical analysis?	Are all important confounding factors/subgroups/differences identified and accounted for?	Were subpopulation identified using objective criteria?	Total number of “yes”
Layton et al. (2018)	Yes	Yes	Yes	Yes	Yes	Not applicable	Not applicable	Yes	No	Not applicable	6
Paulke-Korinek et al. (2013)	Yes	Yes	Yes	Yes	No	No	Not applicable	Yes	No	Not applicable	5
Loughlin et al. (2012)	Yes	Yes	Yes	Yes	Yes	Yes	Not applicable	Yes	No	No	7
Belongia et al. (2010)	Yes	Yes	Yes	Yes	No	Yes	Not applicable	Yes	No	No	6
Oberle et al. (2010)	Yes	Yes	Yes	Yes	No	Yes	Not applicable	Yes	No	No	6
Hua et al. (2009)	Yes	Yes	Yes	Yes	No	Yes	Not applicable	Not applicable	No	No	5
Geier et al. (2008)	Yes	Yes	Yes	Yes	No	No	Not applicable	No	No	No	4

It was not possible to assess risk of bias of the studies described in the package insert of the pentavalent vaccine because some information pertaining to these studies could not be accessed (RotaTeq, 2017). According to a systematic review (Soares-Weiser et al., 2012), one of the clinical studies (Phua et al., 2009) fulfilled all the assessment criteria for risk of bias and, therefore, had minimum bias risk. Another clinical trial (Salinas et al., 2005) had risk of bias for allocation and reporting of selective outcomes, whereas one study (Phua et al., 2005) had risk of bias for most of the criteria assessed.

### Results of Outcome Evaluated and Quality of Evidence

None of the studies reported the rate of discontinuation of the vaccination schedule. Some studies were not included in the meta-analysis because they failed to report data on the incidence of Kawasaki disease.

**Figure 2:**
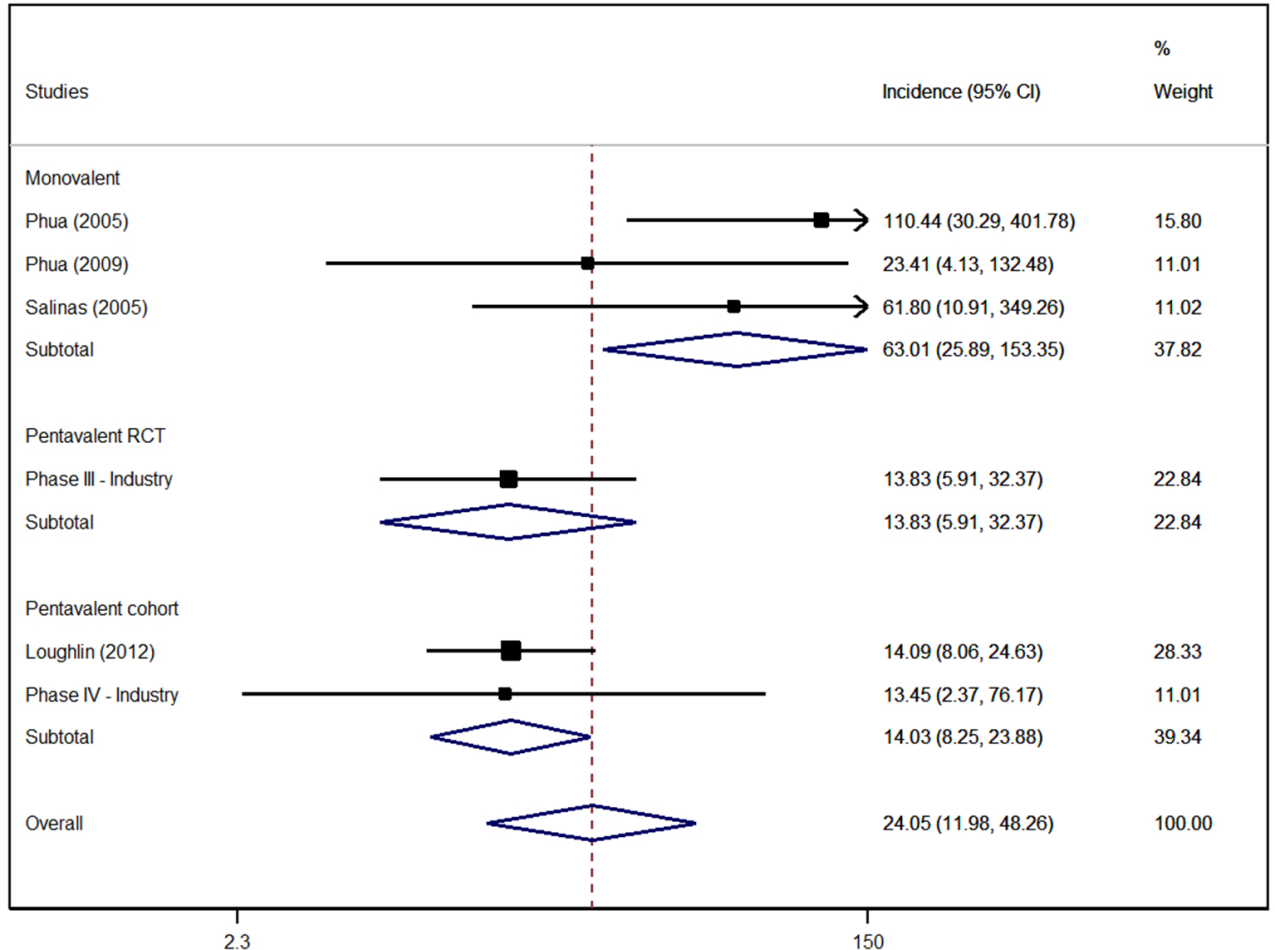
Incidence of Kawasaki disease (per 100,000 children) due to use of rotavirus vaccines.

The meta-analysis revealed a rare incidence of cases of Kawasaki disease, with 24 cases per 100,000 vaccinated children for both vaccines (95% CI = 11.98–48.26). No differences between vaccines were found for incidence of the adverse effect (relative risk = 1.55 95% CI = 0.41–5.93) (**Figure 2**).

**Figure 3:**
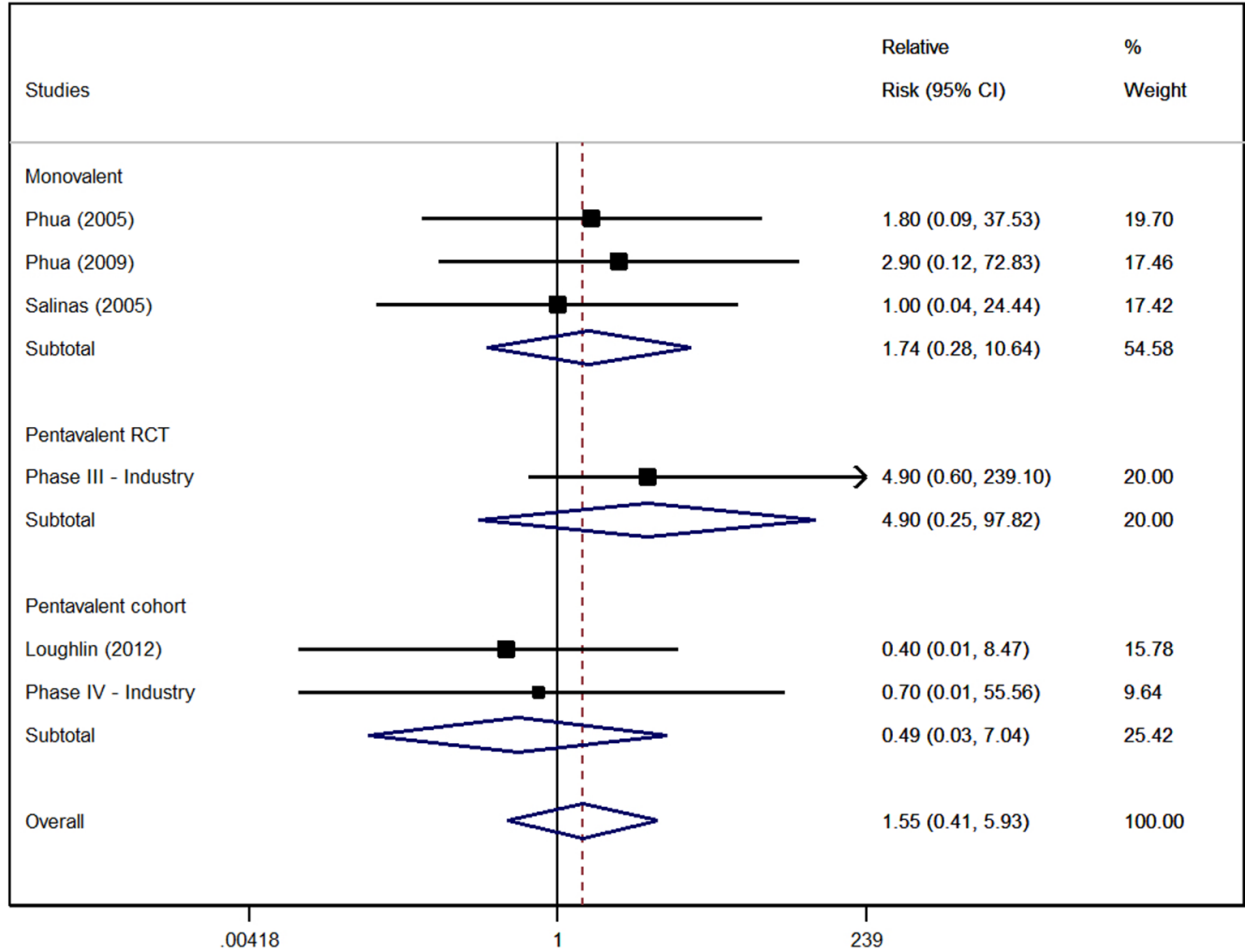
Risk of developing Kawasaki disease due to use of rotavirus vaccines.

The risk of developing Kawasaki disease in the group of children receiving the vaccines did not differ to the comparator group, where no statistical difference was found between them. No heterogeneity was observed among the studies (**Figure 3**). However, the quality of the evidence according to the GRADE criteria for this outcome was considered low for both the vaccines, due to the high risk of bias, and imprecision in the results obtained.

Subgroup analyses were performed between studies conducted in the West (Salinas et al., 2005; Loughlin et al., 2012) and Asian-Pacific (Phua et al., 2005; Phua et al., 2009) countries. There was a higher incidence of disease in Asian-Pacific (57 per 100,000, I^2^ = 49.4%) compared to Western (23 per 100,000, I^2^ = 60.5%) countries. Subgroup analyses were not carried out for age, gender, and race due to the absence of information in the eligible studies.

## Discussion

### Summary of Findings and Their Interpretation With the Available Literature

The findings of this review showed that the occurrence of Kawasaki disease is rare in children that received the monovalent or pentavalent vaccines. In addition, the risk of having the disease in the children receiving the vaccines against the rotavirus did not differ to comparator group.

A total of 13 publications reporting the frequency of the Kawasaki disease were included, the majority of which were conducted in the United States of America and Asian countries. The studies reported information on both the monovalent and pentavalent vaccines, where only one case report was found on the Lanzhou lamb rotavirus, marketed solely in China.

In general, the studies concluded that there was no increased risk or causal relationship of the adverse effect with use of the vaccines. However, some publications (Geier et al., 2008; Hua et al., 2009; Belongia et al., 2010) reported the need for monitoring the use of these vaccines in order to gather further information on their safety.

The rate of discontinuation of the vaccination schedule was not reported in included studies. This occurred either because the cross-sectional studies described the adverse effects reported in the databases or because the longitudinal studies did not bother to collect this outcome, important for endemic control programs.

The studies that involved the collection of information in databases were not included in the meta-analysis owing to the absence of data enabling the incidence of the adverse effect to be determined. The authors of these studies highlighted some shortcomings in the collection of the information in the databases used. The difficulty establishing a causal relationship between Kawasaki disease and the use of the vaccine stems, in part, from uncertainties regarding the information reported in the databases (Paulke-Korinek et al., 2013), and from the concomitant use of the rotavirus vaccine with vaccines against diphtheria, tetanus, and pertussis and/or pneumococcus, among others (Hua et al., 2009; Oberle et al., 2010; Layton et al., 2018), demonstrating that other vaccines could also cause the disease.

In the researched literature, we found studies that evaluated the use of vaccines in children, such as measles, mumps, rubella, and varicella (MMRV) vaccine; measles, mumps, and rubella (MMR) vaccine (Holman et al., 2010); and pneumococcal vaccine (Park et al., 2011) and observed no association between vaccination and Kawasaki disease. We found no studies that evaluated the risk of Kawasaki disease due to combination of the rotavirus vaccine with any other vaccine (diphtheria, tetanus, and pertussis and/or pneumococcus).

In the present study, higher incidence of disease in Asian-Pacific countries compared to Western countries was observed. These results agree with the literature that have reported higher incidence of the disease in children of Asian and Japanese ethnicities (Holman et al., 2010; Park et al., 2011). However, the genetic characteristics of those children susceptible remain only partially elucidated (Principi et al., 2013). Subgroups analysis for age, gender, and race were not performed due to the absence of information in the eligible studies.

In addition, some studies failed to report how Kawasaki disease was diagnosed or reported, such as the studies based on data from the VAERS (Geier et al., 2008; Hua et al., 2009). The authors noted that the rates of notifications obtained by the system could not be interpreted as real, given possible under-reporting of the adverse effects, which in turn may have been largely due to difficulties confirming the disease diagnosis and to the way this is recorded on the systems.

The revision of the package insert of pentavalent vaccine carried out in 2007, which included Kawasaki as a serious adverse effect, which led to an increase in the number of notifications of this disease (Hua et al., 2009; Hua et al., 2010; Oberle et al., 2010). The study of Hua et al. (2009) noted a rise in the number of annual cases from 0.65 to 2.78 per 100,000 children less than 5 years of age followed for up to 30 days post-vaccination, after amendment of the insert.

### Assessment of Study Validity: Limitations and Strengths

The difficulty in the description of the information registered on the systems/databases reported by the studies is a limitation of the present investigation. Measurement bias was also observed, owing to problems concerning the description of method of disease diagnosis (or absence of standard for diagnosis), which was not reported by some of the studies. This might be explained, in part, by difficulties studying Kawasaki disease, given the lack of a standard case definition coupled with insufficient knowledge of etiology.

The lack of a standard for diagnosing the disease can lead to the inherent underreporting of data on these databases. However, this occurred mainly in cross-sectional and adverse event report studies, which were not included in the meta-analysis.

In cases where the information on some RCTs proved inaccessible, the diagnostic criteria for the disease were described as “not reported.” However, the design of this type of study ensures, with some confidence, that the adverse effect was measured rigorously. With regard to the observational studies, it is important to emphasize that the results found in the meta-analysis do not imply causation, since there is always the possibility of residual confounding in these studies.

Despite the broad search of scientific articles in several different databases and exhaustive attempts to obtain the missing information for some of the selected studies, few studies contained all the information required, as exemplified by some information from clinical trials provided by the systematic review and the absence of details in some studies described (such as number of doses of the rotavirus vaccine given prior to onset of Kawasaki disease, time to onset of Kawasaki disease after rotavirus vaccination, use of concomitant vaccinations, among others), hampering the analysis of bias risk. This study did not search specific databases for Asian and Japanese children, in whom the literature indicates a possible higher prevalence of the disease.

Notwithstanding, this systematic review and meta-analysis pooled the incidence of Kawasaki disease due to use of the rotavirus vaccine. The results exhibited no heterogeneity across studies, partly due to the high number of participants. Moreover, the method employed in this study was rigorous with explicit eligibility criteria and a broad search. The assessment of the quality of evidence was based on an independent assessment of bias risk, imprecision, consistency, indirect measures, and publication bias.

### Clinical Implications and Future Perspectives

The results of the present study suggest no association of Kawasaki disease with the use of monovalent and pentavalent vaccines. However, these results should be interpreted with caution due to low quality of the evidence from the studies included in the meta-analysis. The difficulty in conducting studies that did not associate rotavirus vaccine with other vaccines is due to the age of children who are receiving many of them. This difficulty finds robust epidemiological evidence to associate this adverse effect with the rotavirus vaccine. Then, future studies should be concerned with minimizing these biases.

The study also showed that observational studies assessing the incidence and causality of this adverse effect in the literature are scarce. This information underscores the importance of the use of vaccines, in view of the risks of contamination by the rotavirus in children under 5 years and the efficacy of these in preventing the infection (Tate and Parashar, 2014; Kollaritsch et al., 2015; Santos et al., 2016). However, the literature studied highlights the need for notification of adverse effects related to the vaccines in order to ensure continuous monitoring of these and other possible effects associated with the vaccines.

Although the results indicate low incidence of Kawasaki disease in children that used the rotavirus vaccines, it is important that health professionals and society at large report these and other adverse effects associated with the vaccines, rendering notification common practice, thereby contributing to the monitoring of safety data on the use of vaccines. This study reports the evidence on risk of Kawasaki disease with the use of rotavirus vaccines, and this finding can help guide health professionals in clinical decision-making.

## Conclusion

The results of the present study indicate that the monovalent and pentavalent vaccines were associated with a low incidence of developing Kawasaki disease, showing no association with this serious adverse effect. However, further studies involving larger samples are needed to confirm these findings.

## Author Contributions

NM is the principal investigator, participated in all stages of the study, and oversaw the writing of the manuscript. CB and MS are the project managers and co-investigators, were involved in study selection and extraction and statistical analysis, and contributed to the writing and revision of the manuscript. LL, MM, FDF, and SB-F are co-investigators, took part in study selection and extraction and contributed to the writing and revision of the manuscript. All authors read and approved the final manuscript.

## Funding

This project is funded by the governmental Program Graduate Education Institutions-PROSUP-CAPES/UNISO.

## Conflict of Interest Statement

The authors declare that the research was conducted in the absence of any commercial or financial relationships that could be construed as a potential conflict of interest.

The handling editor is currently organizing a Research Topic with MS, LL, SB-F, and CB and confirms the absence of any other collaboration.

## Abbreviations

CINAHL, Cumulative Index to Nursing and Allied Health Literature; GRADE, Grading of Recommendations Assessment Development, and Evaluation; FDA, Food and Drug Administration; WHO, World Health Organization; PRISMA, Preferred Reporting Items for Systematic Reviews and Meta-Analyses; RCT, randomized clinical trials; CENTRAL, Cochrane Central Register of Controlled Trials; PROSPERO, International Prospective Register of Systematic Reviews; VHL, Virtual Health Library; ICTRP, International Clinical Trials Registry Platform.
