# Supplementary material for: Kawasaki Disease and the Use of the Rotavirus Vaccine in Children: A Systematic Review and Meta-Analysis
Source: Front Pharmacol. 2019 Sep 24;10:1075. doi: 10.3389/fphar.2019.01075 (PMC6768949; doi:10.3389/fphar.2019.01075)
Supplement: Supplementary file 1 [file DataSheet_1.docx]

**The Cochrane Central Register of Controlled Trials (CENTRAL)**

# Kawasaki Disease **(2)**

**MEDLINE**

# 1. Rotavirus Vaccines.mp. or exp Rotavirus Vaccines/ 1882

# 2. Vaccines, Rotavirus.mp. or exp Rotavirus Vaccines/ 1585

# 3. 1 or 2/ 1887

# 4. adverse effects.mp./ 83303

# 5. side effects.mp./ 182454

# 6. undesirable effects.mp./ 1750

# 7. injurious effects.mp./ 6789

# 8. 4 or 5 or 6 or 7/ 266193

# 9. 3 and 8/ **49**

**EMBASE**

# 1. Rotavirus Vaccines.mp. or exp Rotavirus vaccine/ 4061

# 2. exp Rotavirus vaccine/ or Vaccines, Rotavirus.mp./ 3854

# 3. 1 or 2/ 4063

# 4. adverse effects.mp. or exp adverse drug reaction/ 454471

# 5. side effects.mp. or exp side effect/ 539588

# 6. exp adverse drug reaction/ or undesirable effects.mp/ 357006

# 7. injurious effects.mp./ 6176

# 8. 4 or 5 or 6 or 7/ 740518

# 9. 3 and 8/ **455**

**CINAHAL**

# 1. "Rotavirus Vaccines" /335

# 2. "Rotavirus Vaccines") OR "Vaccines, Rotavirus" / 288

**#** 3. 1 OR 2/ 338

#4. "Adverse Drug Event+") OR "adverse effects" / 219.888

# 5. "side effects" / 13.233

# 6. "injurious effects" / 13

# 7. 4 OR 5 OR 6 / 227.337

# 8. 3 AND 7 / **110**

**WEB OF SCIENCE**

# 1. Rotavirus Vaccines/ 3395

# 2. Vaccines, Rotavirus/ 3395

# 3. 2 or 1/ 3395

# 4. adverse effects/ 170.754

# 5. side effects/ 237.782

# 6. undesirable effects/ 12.714

# 7. injurious effects/ 2.601

# 8. 4 or 5 or 6 or 7/ 400.676

# 9. 8 and 3/ **54**

**HEALTH STAR**

# 1. Rotavirus Vaccines.mp. or exp Rotavirus Vaccines/ 1.577

# 2. Vaccines, Rotavirus.mp. or exp Rotavirus Vaccines/ 1.359

# 3. adverse effects.mp./ 61.805

# 4. side effects.mp./ 61.805

# 5. undesirable effects.mp./1.134

# 6. injurious effects.mp./ 220

# 7. 1 or 2/ 1.581

# 8. 3 or 4 or 5 or 6/ 192.000

# 9. 7 and 8/ **52**

**LILACS**

# Rotavirus Vaccines or Vaccines, Rotavirus and adverse effects or side effects or undesirable effects or injurious effects/ **11**

**SCOPUS**

# Rotavirus Vaccines and Kawasaki Syndrome or Lymph Node Syndrome Mucocutaneous or Kawasaki Disease/ **76**

**Clinical trial.gov**

#Rotavirus Vaccines and Mucocutaneous Lymph Node Syndrome/ **1**

**International Clinical Trials Registry Platform**

# Rotavirus Vaccines and Mucocutaneous Lymph Node Syndrome/ **0**

**Brazilian Digital Library of Thesis and Dissertations**

“Rotavirus Vaccines” and “Kawasaki Syndrome” or “Lymph Node Syndrome Mucocutaneous” or Kawasaki Disease/ **0**

**ProQuest Dissertation & Theses Database**

Rotavirus Vaccines and Kawasaki Syndrome or Lymph Node Syndrome Mucocutaneous or Kawasaki Disease/ **0**

**Thesis and Dissertation Catalog of Coordenação de Aperfeiçoamento de Pessoal de Nível Superior (CAPES)**

Rotavirus Vaccines and Kawasaki Syndrome or Lymph Node Syndrome Mucocutaneous or Kawasaki Disease/ **0**

**Google Scholar**

Rotavirus Vaccines and Kawasaki Syndrome or Lymph Node Syndrome Mucocutaneous or Kawasaki Disease/ **300**
